# Vaccination barriers and drivers in Romania: a focused ethnographic study

**DOI:** 10.1093/eurpub/ckac135

**Published:** 2022-11-23

**Authors:** Eve Dube, Adriana Pistol, Aurora Stanescu, Cassandra Butu, Sherine Guirguis, Oana Motea, Anca Elvira Popescu, Alexandra Voivozeanu, Miljana Grbic, Marie-Ève Trottier, Noel T Brewer, Julie Leask, Bruce Gellin, Katrine Bach Habersaat

**Affiliations:** Direction des risques biologiques et de la santé au travail, Institut National de Santé Publique du Québec, Québec, QC, Canada; Axe maladies infectieuses et immunitaires, Centre de Recherche du CHU de Québec-Université Laval, Québec, QC, Canada; Vaccine Acceptance and Demand, Vaccine Acceptance Research Network, Sabin Vaccine Institute, Washington, DC, USA; Centre for Communicable Disease Surveillance and Control, National Institute of Public Health Romania, Bucharest, Romania; Centre for Communicable Disease Surveillance and Control, National Institute of Public Health Romania, Bucharest, Romania; World Health Organization (WHO) Country Office in Romania, Bucharest, Romania; Common Thread, Bucharest, Romania; World Health Organization (WHO) Country Office in Romania, Bucharest, Romania; World Health Organization (WHO) Country Office in Romania, Bucharest, Romania; World Health Organization (WHO) Country Office in Romania, Bucharest, Romania; World Health Organization (WHO) Country Office in Romania, Bucharest, Romania; Direction des risques biologiques et de la santé au travail, Institut National de Santé Publique du Québec, Québec, QC, Canada; Department of Health Behavior, Gillings School of Global Public Health and Lineberger Comprehensive Cancer Center, University of North Carolina, Chapel Hill, Chapel Hill, NC, USA; Susan Wakil School of Nursing and Midwifery, Faculty of Medicine and Health, University of Sydney, Sydney, NSW, Australia; Vaccine Acceptance and Demand, Sabin Vaccine Institute, Washington, DC, USA; Vaccine-Preventable Diseases and Immunization, World Health Organization (WHO) Behavioural and Cultural Insights unit and WHO Europe, Copenhagen, Denmark

## Abstract

**Background:**

In 2016–18, a large measles outbreak occurred in Romania identified by pockets of sub-optimally vaccinated population groups in the country. The aim of the current study was to gain insight into barriers and drivers from the experience of measles vaccination from the perspectives of caregivers and their providers.

**Methods:**

Data were collected by non-participant observation of vaccination consultations and individual interviews with health workers and caregivers in eight Romanian clinics with high or low measles vaccination uptake. Romanian stakeholders were involved in all steps of the study. The findings of this study were discussed during a workshop with key stakeholders.

**Results:**

Over 400 h of observation and 161 interviews were conducted. A clear difference was found between clinics with high and low measles vaccination uptake which indicates that being aware of and following recommended practices for both vaccination service delivery and conveying vaccine recommendations to caregivers may have an impact on vaccine uptake. Barriers identified were related to shortcomings in following recommended practices for vaccination consultations by health workers (e.g. correctly assessing contraindications or providing enough information to allow an informed decision). These observations were largely confirmed in interviews with caregivers and revealed significant knowledge gaps.

**Conclusions:**

The identification of key barriers provided an opportunity to design specific interventions to improve vaccination service delivery (e.g. mobile vaccination clinics, use of an electronic vaccination registry system for scheduling of appointments) and build capacity among health workers (e.g. guidance and supporting materials and training programmes).

## Introduction

Measles is a serious and highly infectious disease, one of the leading causes of morbidity and mortality among young children globally, that can be prevented by vaccination.^1^ Measles is characterized by an exclusively human reservoir and interhuman transmission^2^ and a high level of population immunity is required to prevent transmission and outbreaks.^3^

Despite the European Vaccine Action Plan^4^ goal to eliminate measles by 2020, over 120 000 measles cases were reported between November 2018 and October 2019 in the Region.^5^^,^^6^ In Romania, 17 533 measles cases and 64 deaths occurred between 1 January 2016 and 1 July 2019.^8^ Most cases (77%) were individuals eligible for vaccination, but incompletely vaccinated (one dose instead of two doses).^7^

From 1999 to 2008, the vaccination coverage for the second dose of the measles-containing vaccine reported in Romania was above 95%. However, the coverage decreased to 81% receiving two doses in 2018.^9^ The vaccination coverage from the official Romanian statistics refers only to people registered in the healthcare system with birth certificates and medical records. It is difficult to ascertain how may children are not registered in Romania. It is thus difficult to assess the exact vaccine coverage among communities experiencing disadvantage. In 2013, World Health Organization reported a total of 45.7% of children in disadvantaged communities that have not completed a full immunization scheme with half of them completely unvaccinated.^10^ Other studies have shown lower vaccine uptake rates among disadvantaged communities in Romania.^11–13^

To better understand the contributing causes of the last measles outbreak, in 2017, a survey was conducted with a representative sample of ∼700 caregivers of confirmed and probable measles cases.^14^^,^^15^ Findings pointed to some important service and systems-related challenges, such as costs, lack of information and lack of supply. Findings of other qualitative studies have shown that low vaccine confidence was a barrier to vaccine uptake in Romania.^16^^,^^17^

Romania is a middle-income country with about 20 million people living in 42 counties and benefiting from a universal healthcare system with state-financed primary, secondary and tertiary healthcare. Primary care is mainly delivered through general practitioners (GPs) who usually practice in clinics, always with practice nurse/s.^10^^,^^18^

Vaccinations in the national programme are provided free of charge and vaccines are not mandatory. GPs and nurses are the main providers of vaccination and receive a small financial compensation from the government for each vaccination consultation, in addition to a general payment per patient.^19^ Some other health workers can also be involved in immunization, such as social workers, Roma representatives, Roma health mediators and community nurses. While these professionals do not immunize patients, they play a key role in enhancing vaccine acceptance in communities by facilitating access to health services and by reaching parents of unvaccinated children to provide tailored vaccination information.

In this context, the aim of our study was to better understand the vaccination experience of caregivers and health workers in communities experiencing disadvantage in order to inform the development of interventions to increase vaccination coverage.

## Methods

Data were collected using focused ethnography (FE).^20^ Ethnography is an applied research methodology that captures social meanings and ordinary activities of people by observing and taking part in their everyday life, while FE is targeted to a distinct issue or shared experience in sub-cultures and specific settings.^20^ FE is used in health research to discover how people integrate health beliefs and practices into their lives, to understand the meaning that members of a subculture or group assign to their experiences and to study the practice of medicine as a culture.^20^^,^^21^ FE helps understanding the participants’ perspectives—in our case, the vaccination experience of health workers and caregivers in communities experiencing disadvantage via interviews, but does not only rely on self-reports and includes also direct observations—in our case of health workers’ practices and patient–health worker interaction.

The Tailoring Immunization Programmes (TIP) approach guided this study. TIP is a process for exploring issues with immunisation in a community and addressing them to improve uptake. It uses the Behaviour Change Wheel as a theoretical process to guide change, which includes understanding the way capability (C), opportunity (O) and motivation (M) affect behaviour Change Wheel process to immunization (Supplementary material).^22^^,^^23^ TIP includes three factors from the COM-B model (B): capability (C), opportunity (O) and motivation (M). TIP values participatory research and stakeholders were involved all aspects of the project, from the conception of the study protocol, the identification of sites for data collection to the interpretation of the findings to the final workshop.

### Recruitment

Purposive sampling was used to identify regions of Romania to recruit clinics in four districts of Romania.

Communities were identified using the national criteria for marginalization utilized in the Atlas for Marginalized Communities of the Romanian Government: ‘communities with a population aged 15–64 with maximum 8 grades of education, people with disabilities or chronic diseases, children aged 0–17, people 15–64 of age not involved in formal labour market or education, life in dwellings with no electricity, overcrowded dwellings and lack of house ownership’.^24^ The National Institute of Public Health and the National Society of Family Medicine, developed a list of potential practices in each region assessing measles vaccine uptake for the year prior to the study. Low uptake clinics were defined as those with a rate below 60% of patients vaccinated with two doses of the measles vaccine while high uptake clinics were those with a rate of above 90%. Contact was made via email or mail with a signed letter from both institutions. A subsequent in-person visit was conducted to practices that expressed interest in participating to explain the study. To capture a variety of experiences and approaches to vaccination different criteria were used to select participating practices. Two practices [one high coverage (>90% two doses) and one low coverage (<60% two doses)] were selected within the same region. We also ensured to include urban/rural practices and at least one practice that worked with a community nurse and another one with a Roma health mediator. In addition, to be selected, all practices needed to:

- Serve communities experiencing disadvantage (at least half of the clientele).
- Serve a significant number of young children under 5 years old.
- Have a contract with the county public health authority to offer immunization as part of the publicly funded programme.

### Procedures and measures

Non-participant observations and qualitative interviews were conducted. The observations focused on an objective assessment of vaccination consultation practices, interactions and the physical environment (e.g. accessibility, waiting time and atmosphere) and professional–carer interactions, whereas the interviews focused on people’s perceptions. Data collections occurred during February–April 2019 and were conducted by three Romanian research assistants who received a 1-day workshop training in qualitative research methodology, vaccinology and best practice for vaccination consultations. They were experienced in qualitative research and had a background in social science or public health. Interviews with health workers were recorded, transcribed verbatim and verified again with audio reference. Notes were taken during interviews with caregivers. Written notes in the observation grid were summarized after every observation session.

### Observations

Observers were not blinded to coverage rate of clinics. Observations were semi-structured using an observation grid and field notes. The grid (Supplementary appendix S1) enabled researchers to track whether recommended behaviours for vaccinators were being routinely implemented and to document relevant activities, statements and observations in relation to the vaccination consultation. It was developed based on guidance and best practices for vaccination consultations and caregiver–health worker interaction.^25^

### Interviews

In each clinic, 2–5 qualitative semi-structured interviews explored the perceptions of GPs, nurses, mediators and other health workers (clinic social workers, Roma representatives, Roma mediators and pharmacists). Participants were asked questions to explore their opinions and perceived role in childhood vaccination, their sources of information, possible continuing education received, the challenges they face when interacting with caregivers or people in their community, the strategies and skills they use and their need for tools and support to address these challenges.

When possible after the consultation, consenting caregivers were asked a few questions to explore their vaccination experience and assess whether it met their needs and expectations, including as regards health worker responses to their questions or concerns, their information needs and preferences and any possible (better or worse) vaccination experiences in other settings. They were also asked about peer support and community values as regards vaccination. In one setting, interviews were conducted with people in the community to obtain perspectives from people who were not in clinics and thus had not necessarily made a positive choice to vaccinate.

### Workshop

In August 2019, a workshop was held with members of the research team and Romanian stakeholders to discuss the study’s findings. The most necessary, feasible and actionable interventions to address these barriers were discussed in small group work using the TIP approach.^22^

### Data analysis

Stratified descriptive statistics for low and high measles vaccine coverage clinics were generated. The closed-ended observations (tick boxes) for each of the main categories of the observation grid were coded as 1 (present, tick box checked) or 2 (absent, tick box not checked). Comparisons of observations according to clinics’ measles vaccine coverage (high vs. low) were performed. Missing data were excluded.

Interviews with health workers were recorded, transcribed verbatim and the transcriptions were translated into English by a professional translator. Written notes in the observation grid were summarized and entered into a Word document after every observation session. First, data were coded inductively using a constant-comparative and concept-development approach on emerging themes.^26^ Second, the inductive categories were allocated to components adapted in the TIP approach.^22^

## Results

We conducted 148 observations in 8 clinics and 161 interviews: 10 with GPs, 9 with nurses, 6 with other health workers, 99 short interviews with caregivers and 37 in-depth interviews caregivers, health workers and other key stakeholders. The characteristics of participating clinics are shown in table 1. Clinics had a median flow of 2165 patients.

**Table 1:** Description of the sample

Clinics	Measles vaccination coverage	Setting	Patients attending the clinics		Observations of vaccination consultation conducted in the clinic		Interviews with health workers		Exit interviews with caregivers	
			*n*	%	*n*	%	*n*	%	*n*	%
1	High	Urban	2729	14.95	43	29.05	1	3.85	31	28.44
2	Low	Rural	∼1700	9.31	6	4.05	4	15.38	11	10.09
3	High	Urban	2700	14.79	8	5.41	2	7.69	6	5.5
4	Low	Rural	2131	11.67	11	7.43	1	3.85	10	9.17
5	High	Urban	∼2000	10.95	32	21.62	3	11.54	22	20.18
6	Low	Rural	1900	10.41	15	10.14	2	7.69	9	8.26
7	High	Rural	∼2200	12.05	20	13.51	6	23.08	10	9.17
8	Low	Urban	∼2900	15.88	13	8.78	7	26.92	10	9.17
	**Total**	**8**	**∼18 260**	**100**	**148**	**100**	**26**	**100**	**109**	**100**

### Key insight from observations of vaccination consultations

**Figure 1:**
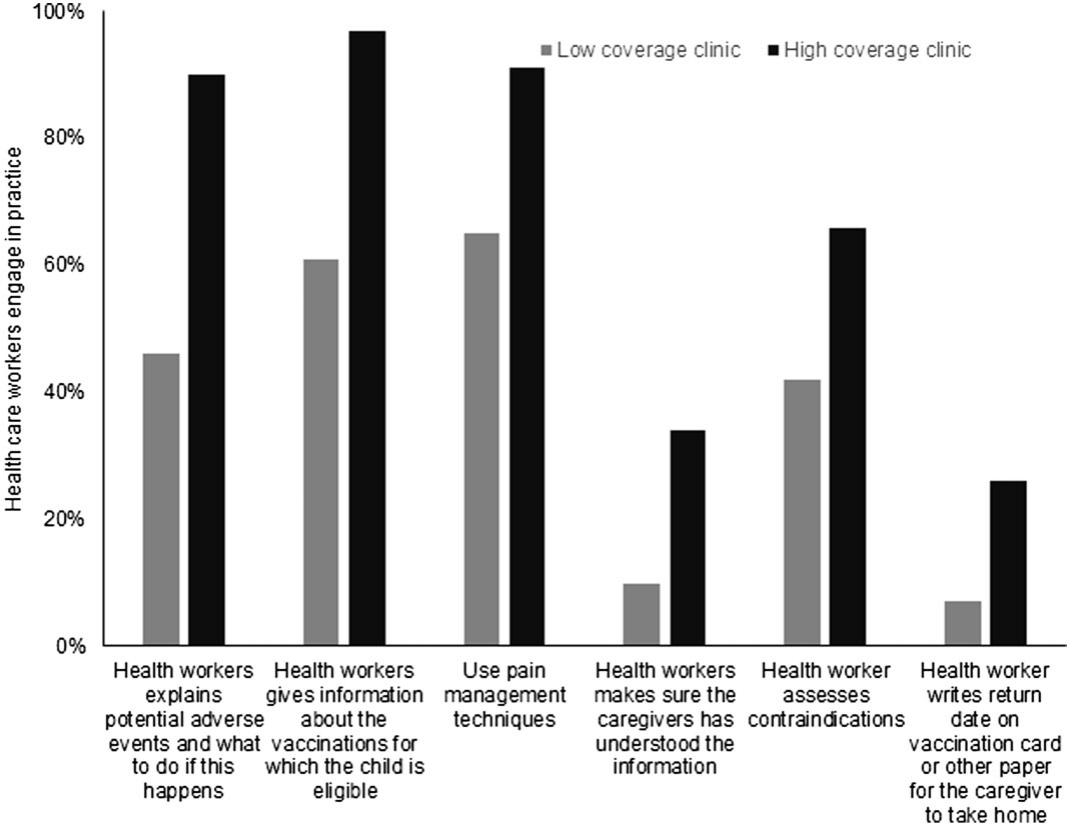
Differences between high and low measles coverage clinics

In general, interactions during consultations were positive and respectful both in high and low-coverage clinics, except one low-coverage clinic (figure 1). Contraindications were assessed at a rate of 33% at the beginning of the consultation in low-coverage clinics and 66% in high-coverage clinics. However, many false contraindications were observed (e.g. not recommending the vaccination of a child who took antibiotics in the month prior to the consultation).

Appropriate techniques were used for vaccine administration in the majority of observed consultations. Clinics with high measles vaccination coverage used pain management techniques more frequently than clinics with low coverage. Appropriate information on potential adverse events following immunization was delivered by health workers in most vaccination consultations in clinics with high coverage, but not discussed in clinics with low coverage.

Most had procedures to call back families and schedule appointments; however, these varied between clinics. Observations revealed that substantial time appears to be invested in scheduling and managing appointments for patients. It was observed that many patients did not drop in appointments by not going to the clinic on time, by coming without having an appointment or by not showing to scheduled appointment which created long waiting time for all patients.

### TIP key insights

Drivers and barriers to vaccination are presented according to Capability, Opportunity and Motivation^23^^,^^27^ (quotes are available in the Supplementary material).

#### Capability

Knowledge among caregivers varied, with some being well informed of the diseases and the vaccines, and others having very low level of knowledge. The lack of knowledge was not necessarily associated with negative perceptions of the benefits of vaccines. However, it was observed that caregivers’ knowledge was generally higher in clinics where health workers were proactively discussing vaccination.

Many caregivers seemed to rely on their GP to reach out to them to remind them about each vaccination and did not know which vaccine was given. The lack of practical knowledge in relation to vaccination was particularly evident in caregiver interviews conducted outside of the clinic, in a community who experienced disadvantage.

Fear of adverse events was identified as a barrier to vaccination and many health workers referred to seeing misinformation on vaccination circulating on social media. However, we observed very few consultations where caregivers directly expressed fears and concerns about vaccination.

The low level of literacy and numeracy was identified as a barrier, especially among caregivers from communities experiencing disadvantage. There also seemed to be a lack of adapted tools or guidance from health authorities on how to address low literacy.

Families were often busy meeting their basic needs like access to water, clothes, food, shelter, etc. Without the capacity to meet basic physiological needs, safety and health, needs like vaccination were secondary.

#### Motivation

Most caregivers had positive intentions towards vaccination of their children. Very few cases of vaccine refusals were observed although the frequent postponing of vaccination appointments could be covert with was described by health workers as covert refusals.

A high level of trust in health workers’ advice and recommendations were observed in both high and low measles coverage clinics, and the vast majority of caregivers agreed to vaccinate if recommended by their GP.

In general, caregiviers had a desire to protect children from diseases, and vaccination was perceived as an effective means to do so. However, among some, especially in communities experiencing disadvantage, prevention was of less importance.

#### Opportunity

In general, vaccination was perceived as the norm. However, refusing to vaccinate was not perceived negatively by peers, and social pressure to vaccinate one’s child appeared to be low.

The majority of health workers promoted vaccination. However, few health workers recommended against and conveyed information on vaccination that is not aligned with current recommendations like not eating chocolate, taking a bath or eating fruits with seeds after vaccination.

Observations indicated that in about half of consultations, the health worker does not explain which vaccines are given for which diseases, and no information is provided regarding the return date. Similarly, very few health workers were inviting questions from caregivers during the consultation. The lack of information provision observed during consultations and confirmed in interviews provides a barrier for caregivers in making informed decisions about vaccination.

While some of the health workers were well informed and very knowledgeable about vaccination, we also observed inadequate levels of knowledge regarding best practices on vaccination. It should be highlighted that many health workers indicated that they would appreciate more information and training opportunities on vaccination. Lack of time and/or interest in keeping up to date with vaccination was noted as a barrier, particularly among the health workers that according to the observations needed training the most.

Very few issues regarding access to vaccination services were identified. Clinics were generally geographically close to the communities and opening hours appeared to be sufficient to meet caregivers’ needs.

Most clinics had a procedure to reach out to patients to remind them about vaccination. A diversity of communication strategies was used (e.g. social media, text messages, telephone calls, home visits or asking relatives). It is unclear if these were in fact effective as many caregivers reportedly did not show up anyway.

Many issues around vaccine supplies were observed. No ‘extra’ vaccines can be provided to clinics, which limits the opportunity to vaccinate patients who are coming to the clinic for other medical reasons. A shortage of specific vaccines was also noted.

One potentially major barrier was the lack of identity papers for some children in communities experiencing disadvantage. Health workers mentioned that vaccines in the publicly funded vaccination programme could be administered for free only to people with identity papers.

Vaccination was perceived by some health workers as demanding, especially with regards to administrative tasks (vaccine procurement, registration, etc.). Some health workers mentioned that they lacked financial incentives to support their work related to vaccination.

#### High vs. low measles coverage clinics

Findings suggest that in low coverage clinics, welcoming and pre-vaccination procedures in consultation room (e.g. assessing contraindications, communicating the agenda for the visit to caregiver, building rapport) were less frequently implemented than in high measles coverage clinics. Differences were also observed in relation with how healthcare workers interacted with and communicated information to caregivers (e.g. giving information on diseases prevented by vaccinations, inviting questions and making sure that the caregivers understood the information). Pain management techniques and adequate administration techniques were observed more frequently in high measles coverage clinics compared to low measles coverage clinics. Scheduling the next vaccination appointment and giving information to caregivers on the management of adverse events were also observed more frequently in high measles coverage clinics.

#### Workshop

Eight barriers were identified as priorities for interventions during the workshop (table 2).

**Table 2:** Priority barriers to address, as discussed in the workshop

COM-B factors	Barriers
Capability	Health workers: inadequate knowledge (medical, legislation) among some
	Health workers: inadequate skills (behavioural, administration) among some
	Caregivers: low knowledge (when to go for which vaccination) among many
Opportunity	Access for caregivers in community experiencing disadvantage (physical and social)
	Ineffective systems to call and remind caregivers about vaccination and long waiting time in some clinics
	Vaccination consultations not conducted according to best practice
	Challenges with stock-outs, supply
Motivation	Lack of incentives for health workers to reach vaccination targets and receive training on vaccination

Recommendations included to develop a strategy and plan for capacity building of health workers focusing on (i) guidance and supporting materials and (ii) training programmes. To enhance health workers’ motivation to access and use new supporting materials and to attend trainings, potential incentives, support and requirements should be considered.

To address the opportunity barriers among caregivers in communities experiencing disadvantage, an environmental restructuring intervention based on mobile vaccination clinics was discussed. This intervention should involve communications on the importance of prevention and that vaccination is free and available even without identification papers.

It was agreed that issues related to ineffective scheduling of appointments, long waiting times and nurses spending substantial time calling and reminding caregivers about vaccination need to be addressed through the already existing national immunization registry.

Finally, participants agreed that issues related to vaccine supply and stock-outs should be addressed. In this context, political commitment and investment will be necessary.

## Discussion

The ongoing pandemic of SARS-CoV-2 is a clear reminder of the importance of vaccination to prevent deaths and suffering. When vaccination rates are sub-optimal, vaccine-preventable diseases can spread rapidly. Under- and non-vaccination can be attributed to both issues related to access to health and vaccination services and to lack of vaccine confidence.^28^^,^^29^ Romania is a centralized healthcare system and rural areas are less well served than urban settings which can result in barriers to access to vaccination services for some population groups.^10^ Upstream factors such as enhanced access to health services and alleviation of childhood poverty could play a role in improving vaccination coverage.^10–13^

The interaction between health workers and caregivers remains the cornerstone of vaccine uptake.^30^^,^^31^ One of the main drivers of vaccine uptake is receiving a recommendation for vaccination by a health worker.^32^^,^^33^ Studies consistently have shown that the majority of caregivers look to their child’s health worker for information and advice on vaccine-preventable diseases, vaccines and the recommended schedule.^34^^,^^35^

The findings of this study indicated that many health workers in Romania are not implementing the national and international recommended practices that relate to vaccination consultations to the desired degree.^36^ At the same time, a clear difference in various steps around immunization was found between clinics with high and low measles vaccination uptake. In low measles coverage clinics, suboptimal practices were identified with regards to interaction between healthcare providers and caregivers before, during and after vaccine administration. This indicates that implementing recommended practices may have an impact on vaccination uptake. Both types of clinics served communities experiencing disadvantage, so their clientele could not explain the differences.

Some promising drivers should be drawn upon in the further effort to increase vaccination uptake in Romania. The high trust which caregivers expressed in health workers, and which health workers express in health authorities can be leveraged, and the general perception of vaccination as a social norm is a good foundation to build upon in communications with caregivers. Findings of the workshop suggested that the most necessary, feasible and actionable interventions to enhance measles vaccine coverage should focus on reducing vaccine knowledge gaps for healthcare providers and caregivers (e.g. tailored communication for caregivers, incentives to attend trainings for healthcare providers), upgrading access to services for disadvantage populations (e.g. mobile vaccination clinics), implementing proper recall strategies for missed appointments, having enough vaccine supplies in clinics and using evidence-based best practices in immunization by giving adequate trainings to healthcare providers.

In addition, observers were unblinded to practice coverage rates, leading to a potential for differential ascertainment between practice types.

There are two main limitations to this study. First, as for all qualitative studies, the results are not generalizable to all clinics in Romania. However, this study offers rich insight for the development of appropriate strategies to strengthen vaccination services and correspond with health workers’ and caregivers’ needs and interests. Second, it is possible that the normal procedures in health practices were disrupted because of the presence of the researchers, including the influence of observation on behaviours.^37^ However, strategies to overcome this were applied, including data collection by a single and unobtrusive researcher in each practice and carefully chosen and trained in techniques to minimize disruption. Despite limitations, the findings of this study are congruent with those of the questionnaire survey with a representative sample of measles cases conducted in 2017.^14^ We also have reached data triangulation^38^ through using a variety of methods—the observations were congruent with those of the interviews with different types of participants. This gives us confidence in our findings.

## Ethics

In Romania, there is no official national body providing ethical clearance for this kind of study. A once-only ethical committee was established to ensure ethical review and oversight which consisted of academics from the Departments of Public Health, Family Medicine and Psychology from the University of Medicine Bucharest and Babes Bolyay, University of Cluj. Written consent was sought from all health workers under observation. For observations that took place during clinical encounters, researchers attended the consultation with caregivers’ verbal approval. The practice was compensated via payment made to the GP: 10 h at USD 25 per hour, total USD 250 per GP. The recruitment approach for caregivers was decided in collaboration with the GP in each practice. No incentive was given to caregivers.

## Supplementary data

Supplementary data are available at *EURPUB* online.

## Funding

This study was implemented by the Romanian Ministry of Health and National Institute of Public Health with financial support from the World Health Organization, the Sabin Vaccine Institute and the Vaccine Acceptance Research Network (VARN) (2017–2019).

## Disclaimer

The authors affiliated with the World Health Organization (WHO) are alone responsible for the views expressed in this publication and they do not necessarily represent the decisions or policies of the WHO.

*Conflicts of interest*: None declared.

Key points

- Sub-optimal vaccination can lead to measles outbreaks, as happened in Romania.
- Health workers play a crucial role in enhancing vaccine uptake and acceptance.
- Findings of observations and interviews suggest that many health workers in Romanian primary care practices did not follow recommended practices for vaccination.
- Practices in Romania with low measles vaccination coverage needed guidance and training on best practices for vaccination consultations, vaccine safety and communication.

## Supplementary Material

ckac135_Supplementary_Data

## Data Availability

The datasets generated and/or analysed during the current study are not publicly available due to confidentiality issues but are available from the corresponding author upon reasonable request.
