# Supplementary material for: Vaccination barriers and drivers in Romania: a focused ethnographic study
Source: Eur J Public Health. 2022 Nov 23;33(2):222–7. doi: 10.1093/eurpub/ckac135 (PMC10066483; doi:10.1093/eurpub/ckac135)
Supplement: ckac135_Supplementary_Data [file ckac135_supplementary_data.zip › ckac135_Supplementary_Data/ejph-2021-10-om-0986-File006.pdf]

Supplementary material

Observation grid

Date<br><br>Name of research assistant<br><br>Name of clinic	
General environment of the clinic (geographical location in the community, outdoor environment) *	
Waiting rooms (size, number of chairs, posters, leaflets, general look, number of patients waiting and length of waiting time)*	

Welcome from of reception staff*<br><br>(greeting, non verbal behaviour,<br>response to questions, explanation<br>and instructions)	
Physical environment of the<br>consultation room (observe where the<br>nurse and the physician sit, the<br>caregivers, poster and other leaflet,<br>etc.) *	

*These questions should be filed once for each observation day

FOR EACH VACCINATION CONSULTATION

Welcoming and pre-vaccination procedures in consultation room	*Tick if done during the consultation*<br><br>☐ Health worker initiates the consultation by building rapport (greeting/ using baby name or whatever is culturally appropriate to that setting)<br>☐ Health worker briefly sets agenda for visit<br>☐ Health worker has access to documented information on child's age and vaccination history<br>☐ Health worker assesses contraindications, note if any___________________________________<br><br>***Other observations***<br><br>☐ Observe what the health worker does if the caregiver does not have the immunization record / booklet, if the child is not registered<br>☐ Observe level of privacy of consultation (number of people in the room, interruptions during the consultation, etc.)<br>☐ Observe how the health worker approaches the child

Communication of information – interaction between health worker and caregiver	*Tick if done during the consultation*<br><br>☐ Health worker gives information about the vaccinations for which the child is eligible<br>☐ Health worker explain which vaccines are given against which diseases<br>☐ Health worker invites questions<br>☐ Health worker ask for money or other gift outside of official procedures<br><br>***Other observations***<br><br>☐ Observe how the information is delivered<br>☐ Does the health worker listen and respond to caregivers' questions?<br>☐ How does the health worker respond to possible questions hesitancy or refusal form the parent?
Informed consent process	*Tick if done during the consultation*<br><br>☐ Health worker make sure the caregiver has understood the information

	☐ Health worker has a written procedure for refusal<br><br>***Other observations***
Vaccine administration	*Tick if done during the consultation*<br><br>Who administer vaccines (nurses vs physician)?<br><br>____<br><br>☐ The vaccine given via the recommended route (legs in babies, arms in 12 months and older)<br>☐ Pain management techniques such as distraction, engaging with the child, breastfeeding are used<br>☐ Health worker administer all vaccinations for which the child is eligible?<br>*If not all recommended vaccinations are administered, note reasons*<br><br>☐ Contraindication (note reason provided)____________________________________<br>_______

	☐ Health worker / caregiver concerns around multiple injections in visit<br>☐ Health worker misinterpretation of the schedule<br>☐ Caregiver concern<br>☐ System barriers (lack of vaccine)<br><br>***Other observations***
Post-vaccination procedures	*Tick if done during the consultation*<br><br>☐ Health workers explain potential adverse events and what to do if this happens<br>☐ Health worker explain return date

	☐ Health worker write return date on vaccination card / other paper for the caregiver to take home<br>☐ Health worker record vaccination in the caregiver's vaccination card<br>☐ Health worker record vaccination in the immunization registry<br>☐ Health worker remind the caregiver to bring back the vaccination card<br>***Other observations***
General observations on the vaccination experience	How long was the consultation?<br>____<br><br>How much time did the health worker talk with caregiver before vaccine administration? ________________<br>☐ The length of time was appropriate

	Other observations<br><br>– What communication skills did you observe with respect to non-verbal behavior, temperament, responsiveness to caregiver, response to emotional cues, etc. (e.g., health worker smile, avoid getting angry for something the caregiver did / did not do, thanked or praised the caregiver for vaccination). What were the reactions of the caregiver (e.g. comfort, fear, confidence, etc.)<br>– Are there environmental conditions explaining the vaccination experience? (crowded waiting room, children crying, etc.)
Other notes (if an interview is conducted with the caregiver, note main findings here)	

Appendix 2. Verbatim and notes from observation for the main drivers and barriers to vaccination

COM-B Factors	Sub-factors	Verbatim and notes from observation

Capability	Caregivers' lack of knowledge of what, who, when of vaccination	I: "Did you hear about measles?" C: "Yes, [. . .] I heard it's a tough disease. I heard this cold it's not good for children. I heard that something bad will happen to him [her son]" (*Interview with caregiver in a community experiencing disadvantage)*<br>*"What about the other vaccines (than the one for influenza)? - They were not necessary. She got sick and was well again*." (Interview with caregiver in a community experiencing disadvantage)<br>*I: "Do you know what the vaccines are for?" C: "I know they are good. . ."* (Interview with caregiver in a community experiencing disadvantage)<br>*"Nobody gave us any vaccine. We live here like in the jungle. […] Nobody tells us about vaccines. We are like animals".* (Interview with caregiver in a community experiencing disadvantage).
	Caregivers' fear of adverse events	Interviewer (I): *"Were you afraid that something might happen to him because of the vaccine?"* Caregiver (C): *"Yes. I heard it on TV".* I: *"What did you hear on TV?"* C: *"I heard that if you give this injection something bad will happen".* (Interview with caregiver in a community experiencing disadvantage).
	Low level of literacy among some caregivers	*When asked what doses have been delayed, she can't answer – seems to struggle to remember how old*

		each child is, laughs it off and tells me she has too many. (Notes from an interview with caregiver, community experiencing disadvantage, rural area).
	Vaccination low priority in communities experiencing disadvantage	*C: "*We don't need vaccines, we need paved roads*" (Interview with caregiver, community experiencing disadvantage, rural area)*
Motivation	Caregivers' intention to vaccinate	*The GP mentioned that caregivers usually vaccinate new-borns, but caregivers of older children are more often reluctant to vaccination. However, they don't refuse vaccination. They stop answering their phone or they keep postponing vaccination* (General observation, High coverage clinic, urban area).
	Caregiver trust in health workers	*C:* "Weird information is spread online, I trust the doctor" (General observation, High coverage clinic, urban area)
	Caregivers' perception of prevention	C: *"I only have one little girl. She didn't get any injection since she was born."* I: *"Why didn't she get any?"* C: *"Because she wasn't sick."* (Interview with caregiver in a community experiencing disadvantage).
Opportunity	Health workers' motivation	GP: *"There are some barriers in terms of contraindications for vaccination raised by our colleagues, the specialist doctors. Paediatricians, child neuropsychiatrists. They contraindicate vaccination for various reasons even though the*

		diagnosis they write in the medical letter does not support the contraindication. They put in writing that vaccination is contraindicated, so it's very hard to convince the caregivers that the specialist was not right and, as he or she is not a vaccine provider, the information on immunization may not be properly up to date." (Individual interview, GP, High coverage clinic, urban area).<br><br>*Caregiver noted that their previous GP has never mentioned any additional vaccines and stressed that even the national program ones were described "optional". "I felt like I had to convince the previous GP that we wanted the vaccines, not the other way around*". (Notes from exit interview with a caregiver, High coverage clinic, urban area).
	Caregivers' involvement in vaccination decisions	*There is very little information exchange. The caregiver seems eager to discuss additional vaccines, but the GP does not continue the conversation. The GP offers no information on current vaccines. Briefly advises on potential side effects but does not mention what the vaccine is for. The GP's tone is neutral. She is abrupt in her delivery when discussing [varicella vaccine] but there is very little conversation even on*

		that topic. (Notes from observation, Low coverage clinic, rural area)
	False contraindications	I: *"What are the contraindications to vaccination that you are looking for?"* GP: *"Fever, loose stools, skin rash, if the child is fidgety. […] Antibiotics cannot be administered alongside vaccines."* (Interview with GP, Low coverage clinic, rural area).<br><br>C: *"I went to give him the vaccine, but he is always sick. . . he has a runny nose. And when I go with him, the doctor tells me he won't give him the vaccine".* (Interview with caregiver, Low coverage clinic, rural area).
	Lack of training opportunities for health workers	GP: *"All vaccinating doctors need to get updated at least once a year which I do on my own because I'm interested in the topic, but other colleagues are not. […] There are vaccinology workshop organized by the GP association, but you need to attend and since you don't have to and it's not something that has your interest because that was not how you grew up, then you will never attend and adjust [your practice]. I think that our colleagues have not read the leaflets in*

		qui some time". (Individual interview, GP, High coverage clinic, urban area).
	Health workers' trust in official information	GP: *"The information we sometimes get from the Public Health Department is not enough. For the pneumococcal vaccine, for example, they sent us an email saying that from X date we will start vaccinating. That was it, no catching up protocol, nothing."* (Individual interview, GP, High coverage clinic, urban area).<br><br>GP*: "There is a shortage of information. We do receive the information when we ask for it, I mean the updates. But I can`t say that we get very (pause) detailed information all of the time.* I: *When you want more information, about contraindications or adverse reactions, for example, do you get the information yourself?* GP: *I get my own information, mostly from each package leaflet. So no, I don`t know how it should be done."* (Individual interview, GP, Low measles coverage clinic, rural area)
	Good access to vaccination services, with a few exception	GP: "*We have limited local resources, such as no transportation means for reaching the patients home. (. . .) The commune has 11 villages, the furthest one is*

		17 km from the commune hall. During winter time it is very difficult to reach patients, as I said no car available (. . .). It is very complicated without public transportation or a car, if necessary, from time to time, we are taking the school bus for home visits." (Individual interview, GP, High coverage clinic, rural area).<br><br>*Nurse talking about patients: "They expect it to be free of charge. And when you explain [that it isn`t] they say that they`ve been registered here since they were babies and have never paid. They don`t pay, this is why the hospital is always full, because they can go there for everything. It`s free."* (Notes from observation, Low coverage clinic, rural area).
	Ineffective systems to call and remind caregivers about vaccination	Nurse: *"I invited her for the shot three times. I called the grandmother, the mother, the father. They told me 11 a.m. Meanwhile I had other families calling for appointments, I already had 3 people scheduled for vaccination saying that they might arrive on the same day so I asked them to call back to see who comes when, to avoid having too many at 11 o'clock. Well, I had been waiting for that family, I called them and they said that they wouldn't come because it was*

		raining and they wouldn't go out on that weather. And I said that I thought they weren't coming because something serious had happened. Like a fever, not rain, and that they should have called to let us know. The grandmother replied that she didn't think it is normal to let us know, they come whenever they want, we have to be there during the working hours and have to see them at any time. So I asked them to call when it stopped raining, what else could I have said? The problem is that they said that they wanted the child to grow up a bit to avoid being hurt by the vaccine". (Individual interview, nurse, Low coverage clinic, rural area)
	Vaccine supply issues	GP*: "I would have done a better job if I had the vaccines all the time. This is a huge problem. You cannot be convincing if you don't have the supplies".* (Individual interview, GP, High coverage clinic, urban area).
	Long waiting time in clinics	GP: *"Despite my efforts to schedule them, they keep coming [whenever they want]. For most of them making an appointment means calling half an hour earlier. Generally speaking, it is easier and also practical if things are predictable, and this is why I am happy that today patients with acute illnesses actually*

		called ahead." (Individual interview, GP, Low coverage clinic, rural area)
	Difficulties in getting unregistered children vaccinated	Nurse*: "We have children whose parents don't have ID papers, birth certificates, etc. so neither do their children and we can't even administer the required shots because we can't enter them into the IT system. These are Roma children, born to teenage mothers most often than not."* (Individual interview, Nurse, Low coverage clinic, rural area).<br>GP: *"They hardly show up for the required shots, there is no point in discussing the optional"* (Individual interview, GP, Low coverage clinic, rural area)
	Lack of financial incentives to vaccinate	Nurse: *"Sometimes, I wish we could avoid children and the whole vaccination process, it's such a headache." I: "Why?"* N*: "Well, it's such an issue, writing everything by hand and then putting it all on the system and keeping track, it's hard".* (Individual interview, Nurse, Low coverage clinic, rural area).
