# Supplementary material for: Vaccination barriers and drivers in Romania: a focused ethnographic study
Source: Eur J Public Health. 2022 Nov 23;33(2):222–7. doi: 10.1093/eurpub/ckac135 (PMC10066483; doi:10.1093/eurpub/ckac135)
Supplement: ckac135_Supplementary_Data [file ckac135_supplementary_data.zip › ckac135_Supplementary_Data/ejph-2021-10-om-0986-File007.pdf]

Interview Guide – Health workers
To be tailored for nurses vs GPs

Introduction. Explain the purpose of the interview. Remind about confidentiality. Start the recorder.

1. Please tell me what your role in the clinic is, how many patients are registered in your clinic and for how long you have been practicing here.

2. Please describe your clientele / the community where you work.

3. Describe me a typical day in your clinic (what activities are you doing? how your day is usually organized?).

4. How is vaccination organized in your clinic? By this I mean things like stocking vaccine, cold chain, record keeping and the process with patients, etc.
    Probes: Is there a protocol you work by? If yes, who developed it?
    Who is allowed to give vaccinations in your clinic (nurses, GPs, others)?

5. How do your recall and booking systems work? How do parents know when it is time for vaccination?

6. Do you do opportunistic* vaccination? Why/why not? (*this means if they use every opportunity to vaccinate children who are missing some vaccines, e.g. when the child is consulting for another reason, e.g., mild illness)

7. How do you record vaccination? Do you do routine monitoring to detect undervaccinated? How?

8. How do you plan for number of vaccines needed for routine immunization? And for supplementary immunization, how do you plan for and organize catch up campaigns?
    Probe: What would help them in organizing catch up campaigns in order to reach herd immunity in the community for all vaccines?

9. Do you know what the vaccination rates for your clinic are? For your community? How do you think your clinic compares with other clinic regarding vaccination?

10. How parents are informed that their child needs a vaccine?
    Probe: What are you doing when parents don't come with their child for vaccination?

11. Do you think most of your colleagues are favorable about vaccination (are pro-vaccine)? Are there any vaccines that you are not sure about?

12. How do you see your own role in relation to vaccination? (e.g., to give vaccines to those who ask for it, to recommend vaccination, to push parents towards vaccinating, etc.)

13. When a child attends for vaccination, what happens with parents before the vaccination is given? What information is given?

14. When a child attends for vaccination, what contraindications do you look for?

Probes: Is there a protocol? Are you following it?
How confident are you in doing this? *If not confident* – what would help?
How often are you unsure? What do you do when you are unsure? Why is that?
Do you often refer the child to a specialist? When?

15. Are there any risk related to vaccination for the local children in your area?

16. What do you know about AEFI (adverse events following immunisation)?
    Probes: Where did you learn this?
    What do you think would happen in case of an AEFI following vaccination where the parents believe the event was caused by vaccination? Will you be held responsible? Will anyone support or protect you?
    If not - how would you like to be supported? At what level should the support be? (PCC, Canton, Federation) Why that level?
    Does this situation affect your willingness to vaccinate children whose parents are reluctant? How?

17. How important do you think communicating with parents in the consultation about vaccination is? Why is that?
    Probe: How confident are you in talking to parents about vaccination? Why is that?

18. How often do you see parents who are unsure about vaccinating their child? How often do you see parents who refuse to vaccinate at all? Tell me about these encounters? (Alternative, can you tell me the last parent you saw who was unsure or didn't want to vaccinate. Tell me what happened.)

19. How do you get information about vaccination from outside the practice?
    Probes: How are you informed about changes in schedule, new recommendations, vaccine shortage, etc.?
    Where are you getting your information or who do you consult when you face a difficult question / problem about vaccination?

20. Do you feel sufficiently informed about vaccination? Why? Why not?

21. What would be the most helpful to support your role in vaccination?

22. Do you think most parents are knowledgeable about vaccination? Do you think most parents in your community want to vaccinate their children?

23. What are the main challenges / most difficult things you are facing around vaccination?
    Probe: What are you doing to make things better?

Ask if any other issue / concerns. End recording. Thanks participant.

Interview Guide - caregivers (to be tailored to context)

These interviews will be conducted with caregivers who consulted for childhood vaccination and who are open to answer a few questions. Interviews will be short and done outside the consultation rooms (depending on the context, discussion could occur in waiting rooms, before the consultation or after, during waiting period after the injection).

Questions will be adapted depending if after / before vaccination experience (if research assistant has observed the consultation). General questions about vaccinations could be asked to people in the clinic, even if they are not coming for vaccinations.

Remind about confidentiality, ask if any concerns and questions.

Ask broad open-ended questions depending on the context on:

- Caregivers' attitudes and opinions toward vaccination (i.e., what do you think about vaccines?, do you trust vaccines are safe? effective?, what have you done in the past regarding vaccination?)
- Caregivers' confidence in their vaccination decision (i.e., you have accepted to have your child vaccinated today, why? – you were reluctant to have your child vaccinated, why?, etc.)
- Caregivers' satisfaction with their vaccination consultations (i.e., how was your consultation today?, what questions or concerns were not addressed by the health workers? have you been elsewhere for vaccination? if yes, what your experience in this other setting better/worse and why?)
- Caregivers' needs and preferences about the vaccination of their children (i.e., what would help you to have your children vaccinated?, how would like to be informed about vaccination? what are other parents doing regarding childhood vaccines in your community?)

If possible, record the age of the child (or approximate age), whether it's a caregiver of many children or one, the age (or approximate age) of caregiver, whether the caregiver came alone or with others?
