# Supplementary figures and images for: Vaccination barriers and drivers in Romania: a focused ethnographic study
Source: Eur J Public Health. 2022 Nov 23;33(2):222–7. doi: 10.1093/eurpub/ckac135 (PMC10066483; doi:10.1093/eurpub/ckac135)

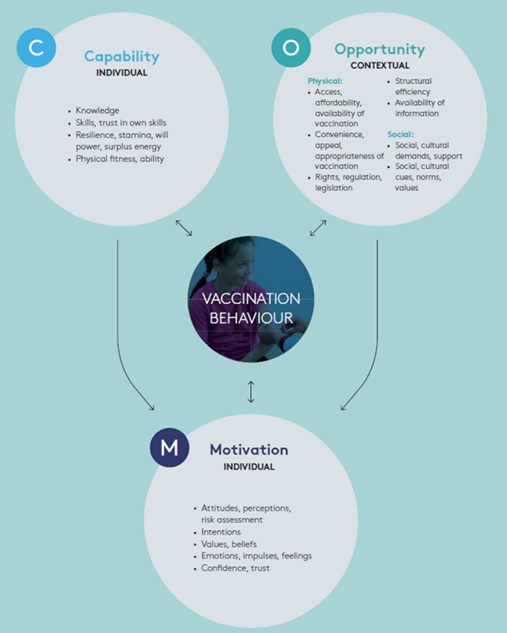

Supplement: ckac135_Supplementary_Data [file ckac135_supplementary_data.zip › ckac135_Supplementary_Data/ejph-2021-10-om-0986-File005.tiff]
